# „Brain doping” substances: prohibited or not in sports?

**DOI:** 10.5114/biolsport.2025.150047

**Published:** 2025-05-14

**Authors:** Andrzej Pokrywka, Olga Surała, Konstancja Grabowska, Marta Przybyła, Dominika Granda, Andrzej Małecki, Raphael Faiss, Marta Nowacka-Chmielewska

**Affiliations:** 1 Department of Biochemistry and Pharmacogenomics, Faculty of Pharmacy, Medical University of Warsaw, Warsaw, Poland; 2 Scientific Team, Polish Anti-Doping Agency, Warsaw, Poland; 3 Department of Nutrition Physiology, Institute of Sport - National Research Institute, Warsaw, Poland; 4 Laboratory of Molecular Biology, Institute of Physiotherapy and Health Sciences, Academy of Physical Education, Katowice, Poland; 5 Center of Research and Expertise in Anti-Doping Sciences, Institute of Sport Sciences, University of Lausanne, Lausanne, Switzerland

**Keywords:** Brain doping, Nootropic, WADA Prohibited List, Dietary supplements

## Abstract

There is increasing interest in the area of brain doping among athletes. The term ‘brain doping’ refers to enhancing mental performance through the non-medical use of specific pharmacological substances, such as drugs and medicines. Despite hundreds of studies, it is challenging to specify well-defined and described substances that could contribute to improving cognitive abilities in sports. Therefore, we systematically reviewed existing scientific literature to identify ‘brain doping’ substances with proven beneficial effects on sports performance. Only 9 research studies, consisting of 7 interventional and 2 case studies, have investigated the current knowledge on substances that have or may affect the brain at the cognitive level in the context of sports. Simultaneously, we reviewed the Polish dietary supplement market to identify active ingredients promoting cognitive functions and examine their properties related to anti-doping rules. Results from the market analysis revealed 34 substances/extracts of plant/mushroom origin and 120 synthetic substances with potential procognitive properties. Among the synthetic substances, 45 were labelled as ‘unclear’, including 19 presumed to ‘meet WADA criteria’. In conclusion, the term ‘brain doping’ currently serves more as a marketing tool than as a concept describing a group of substances with scientifically proven cognitive effects. Substances marketed to consumers as ‘brain doping’ include substances prohibited in sports, as mentioned on the WADA Prohibited List, and those permitted for use by athletes or of unclear status. The open-ended nature of the list of prohibited substances poses challenges in definitively determining the status of many substances found in dietary supplements or medications, which may lead to unintentional violations of anti-doping rules.

## INTRODUCTION

**FIG. 1:**
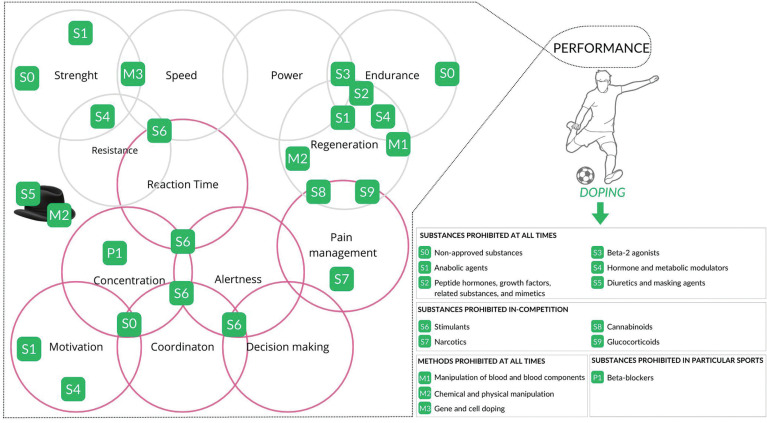
Substances and methods prohibited in sport.

Doping in sports is a recurrent topic since some athletes feel compelled to use forbidden substances or techniques to improve their physical performance. The World Anti-Doping Agency (WADA) was established in 1999 and has been crucial in developing anti-doping rules and policies since 2004 to prevent, deter and detect doping in sports. In that context, doping is defined as a violation of the rules defined in the WADA Code as the main document harmonizing regulation and their application. The use or presence of a forbidden substance in an athlete’s biological sample (blood or urine) thus represents such a violation, and the List of Prohibited Substances and Methods [1] outlines 9 classes of banned substances and 3 classes of forbidden methods that may be linked to performance improvement (Fig. 1). Furthermore, for a substance (or method) to be included in the WADA list it must meet at least two of the following three criteria: 1/it enhances or has the potential to enhance sport performance; 2/it represents an actual or potential health risk to the athlete; 3/it violates the spirit of sport. There is evident room for interpretation in the definition of the spirit of sport, since scientific evidence is sometimes lacking to provide a causal link between the use of a given substance and sport performance. Nevertheless, the Prohibited List is updated annually and covers a wide range of substances and drugs, posing a challenge as the use of numerous medications used to treat diseases may alone or combined result in the presence of metabolites associated with potential anti-doping rule violations. This complexity often leaves athletes and their support personnel in need of assistance to determine the correct status of certain substances of drugs and dietary supplements with the yearly evolving Prohibited List.

To support athletes, various educational initiatives provide information on substances through online databases and apps. The Polish Anti-Doping Agency offers an Anti-Doping Information Hotline, established in 2009, where individuals can inquire about the status of specific substances under anti-doping regulations. Recently, there has been growing interest in ‘brain doping’ among those seeking information.

Despite being a hot topic, a consensus among experts is yet to be reached on the definition of brain doping, also known as neuro-doping. In scientific discussions, the phrase ‘brain doping’ is often used interchangeably with ‘neuroenhancement’ or ‘cognitive enhancement’. These terms generally refer to enhancing mental performance through the non-medical use of specific pharmacological substances, such as drugs and medicines, and supplements in healthy individuals [2–4]. Iglseder (2018) differentiates between pharmacological neuroenhancement and brain doping. Pharmacological neuroenhancement involves the use of legal or non-prescription psychoactive substances, while brain doping explicitly denotes the use of illegal substances or prescription drugs for cognitive enhancement purposes [5]. Some authors define brain doping as the use of non-invasive brain stimulation techniques to enhance athletes’ performance, such as transcranial direct current stimulation (tDCS) and transcranial magnetic stimulation (TMS), but this review does not cover this interpretation [6, 7]. According to Cho et al. (2023), ‘smart drugs’ are a novel form of doping that involves stimulating specific brain regions to enhance sports performance. Brain doping aims to maximize exercise capacity by manipulating signalling systems or secreting neurochemicals to control the brain, enabling the highest extent of exercise possible [8]. Thus, in an antidoping context, one may define ‘brain doping’ as the use of substances or methods to enhance performance by directly or indirectly modulating cognitive capacities, brain activity, or function [9].

Accordingly, in reference to the WADA rules and the prohibited list, there is possibly a loophole with some substances or techniques that could improve the brain function towards enhanced sporting performance while not being on the prohibited list to date. Moreover, it is not always possible to unequivocally determine the status of a substance referred to or promoted as brain doping. This study therefore aimed first to review scientific evidence on brain doping with any related substance or technique, and second to identify compounds marketed and sold as ‘brain doping’ substances and determine their status concerning anti-doping rules.

## MATERIALS AND METHODS

The research comprised two steps: 1/a systematic review of existing scientific literature to identify substances referred to ‘brain doping’ with scientifically proven beneficial effects on sports performance; 2/a review of the Polish dietary supplement market to identify active ingredients promoting cognitive functions and examine their properties related to anti-doping rules.

### Literature search

The present study followed the Preferred Reporting Items for Systematic Reviews and Meta-Analyses (PRISMA) guidelines [10].

### Information sources

The PubMed database was used to identify studies relevant to the review, independently by two researchers, K.G. and M.P. The initial database search was performed in March 2023, with an update on 25^th^ April 2024.

### Search strategy

The following terminology of eight categories was applied: ‘brain doping’, ‘doping for the brain’, ‘neuro-doping’, ‘neuropharmaceuticals’, ‘nootropics’, ‘smart drugs’, ‘cognitive enhancers and doping’, ‘cognition and doping’, ‘new psychoactive substances’. Each category was then connected with four phases – ‘sport’, ‘athletes’, ‘academic sport’, and ‘sport performance’ – for searching in the PubMed database without a year-of-publication restriction.

### Inclusion and exclusion criteria

The inclusion and exclusion criteria are summarised in Table 1. The decision to include an article in the review was made by two authors (K.G. and M.P.). In the event of disagreement, the third author decided (M.C-N.).

**TABLE 1:** Criteria adopted for inclusion and exclusion of publications from the systematic review.

Factor	Inclusion criteria	Exclusion criteria
Database	PubMed	Other sources

Langue	English	All languages other than English

Text	Full-text original articles	Reviews, commentaries, letters, book chapters, conference proceedings

Target population considered by the study	Human subjects	Non-human subjects

Purpose	Focus on the substances that affect the brain at the cognitive level in the context of sports	Focus on studies without drug supplementation by subjects or non-pharmacological cognitive brain stimulation in the context of sport

### Data extraction and analysis

All identified studies were independently reviewed by two authors (O.S. and D.G.), and their results were entered into data tables which were designed before data extraction. The following data were extracted from the interventional studies: authors’ names and year of publication, study design and washout time, dosage, substance and intervention duration, number, sex and age of participants, sport level and cognitive aspects which were assessed. Moreover, we extracted data concerning the cognitive effect of studied substance and/or potential effect on performance (where available) and whether the authors reported any adverse reactions of supplementation. From the case studies the following data were extracted: authors’ names and year of publication, study design, substance, dosage and duration of use, sex and age of the individual, sport characteristic, symptoms at admission and clinical outcome.

### Selection of potentially nootropic substances occurring in dietary supplements

In the third quarter of 2023, we conducted a review of Polish online stores offering dietary supplements. We used various search terms such as ‘dietary supplements,’ ‘cognitive enhancement supplements,’ ‘nootropics,’ ‘brain booster,’ ‘buy online,’ and ‘web shop.’ We analysed a total of 32 online stores for their offerings of dietary supplements, focusing on those promoted with catchphrases such as ‘memory,’ ‘concentration,’ ‘cognitive enhancers’, ‘nootropics,’ ‘brain,’ ‘brain booster,’ and ‘mental performance’. The review was conducted independently by two authors (O.S. and A.P.), and at the end, the search results were compared and tables were jointly prepared.

### Classification of the identified active ingredients

Labels of products categorized as supplements supporting cognitive functions were analysed for active ingredients. Active compounds with potential pro-cognitive effects were assigned to substances of plant/fungus origin (‘botanicals’) or synthetic substances. Botanical supplements were not included in this study due to their complexity – plant/mushroom extracts are multi-component, which makes it difficult to determine specifically which component of the extract produces a pro-cognitive effect. The list of ingredients/supplements from the botanicals category is presented in the supplementary material (Table S1).

### Examination of the properties of the selected substances concerning anti-doping regulation

Synthetic substances were assessed for anti-doping rules and classified into one of the three following categories: prohibited in sports, not prohibited, or having an unclear status. Substances included on the WADA list were classified as ‘prohibited in sports’. The ‘not prohibited’ category included substances that fall into the following criteria: the substance is mentioned on the WADA list as an ‘exception’, or it is included in the WADA Monitoring Program, and/or it is noted as permitted or recommended in sport on the different websites provided by: the U.S. Anti-Doping Agency (USADA), the NSF Certified for Sport (NSF Sport), the National Anti-Doping Agency of Germany (NADA Germany), the Global Drug Reference Online (Global DRO). Global DRO and NADAmed are the Internet databases of banned and allowed drugs in sports, which are intended to enable both athletes and caregivers to obtain easily accessible and rapid information on the doping relevance of medications. Global DRO was brought through a partnership between the Canadian Centre for Ethics in Sport (CCES), Swiss Sport Integrity (SSI), UK Anti-Doping (UKAD), and the United States Anti-Doping Agency (USADA). The Japan Anti-Doping Agency (JADA), Sport Integrity Australia, and Sport Integrity Commission NZ are official Global DRO licensees. In turn, the NADAmed Drug Database contains a selection of frequently prescribed or requested drugs that are approved as medicines or registered as homeopathic medicines in Germany. We also benefited from the ABCD Classification system developed by the Australian Institute of Sport (AIS), which ranks sports foods and supplement ingredients into four groups according to scientific evidence and other practical considerations that determine whether a product is safe, permitted, and effective in improving sports performance [11], as well as the information provided by the organizations of thirdparty certification and testing of dietary and/or food supplements. An example of the latter is NSF International – a global public health organization that facilitates the development of standards, and tests and certifies products for the food, water, and consumer goods industries, and discusses the importance of testing and certifying dietary supplements and what that process entails [12]. The NSF Certified for Sport programme helps athletes, dietitians, coaches, and consumers around the world make safer decisions when choosing sports supplements, functional foods, and personal care products. However, it should be noted that it reduces, but does not eliminate, risk for athletes, because no third-party testing company can test for every possible prohibited substance.

The third category, i.e. ‘unclear status’, included substances that could not be directly assigned to the prohibited or non-prohibited group. In this group, we included those that, according to our knowledge, met the criterion for a doping substance due to a similar chemical structure or similar biological effect(s) to substances mentioned on the WADA list.

### RESULTS

#### Systematic review

**FIG. 2:**
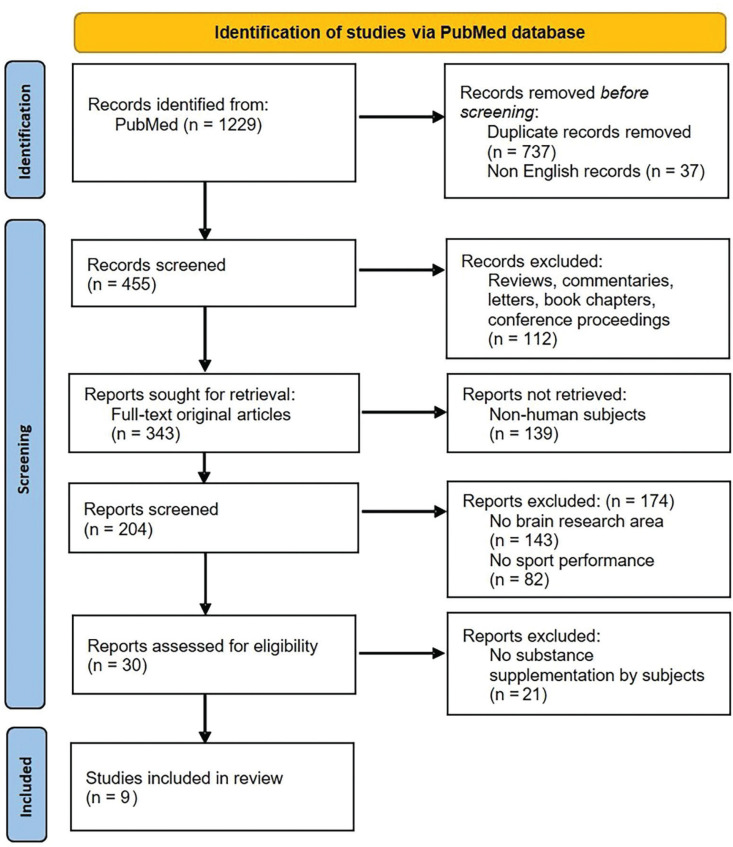
Flow chart showing the procedure of selection of studies included in the review.

1229 records were identified in the initial database search. After the removal of duplicates, 492 records were identified. Only articles written in English were included. All authors then screened the titles and abstracts of 455 articles to select full-text original papers conducted on humans. In this first step of screening, 205 reports were included and full texts were reviewed in order to choose studies addressing both the brain and sports research aspects. Thirty-one studies were selected for evaluation of eligibility. Finally, nine articles that investigated the current knowledge on substances that have or may affect the brain at the cognitive level in the context of sports were included in the review. The selection of papers for this review was performed according to the Preferred Reporting Items for Systematic Reviews and Meta-Analyses (PRISMA) flow chart (Figure 2).

#### Characteristics of included studies and participants

A total of 9 studies were ultimately included in the final analysis, consisting of 7 interventional studies and 2 case studies. The results of these studies were discussed separately for each type of study.

#### Interventional studies

Seven interventional studies (Table 2) were included in the systematic review – all of them were randomized controlled trials with a cross-over study design [13–19]. Included studies were published over the last 22 years: the oldest was published in 2002 (16), the newest in 2021 [19]. Almost every study involved a different substance: tramadol [13], alpha glycerophosphocholine, uridine-5ʹ-monophosphate, and docosahexaenoic acid (DHA) [14], cocoa flavanol [15], choline [16], polyphenols [17], snus [18] and inositol stabilized arginine (19). Only two of the seven studies included female participants [17, 19]; however, their number was too small to conduct a separate analysis in subgroups. In four out of seven included studies, the study group consisted of trained or highly trained athletes [13, 15, 17, 19], while in the remaining three studies, amateurs/recreationally active people were examined [14, 16, 18]. A beneficial effect of the tested substance on at least one of the cognitive domains was detected in 3 studies [13, 17, 19], 3 showed no effect [14–16] and 1 showed a negative impact on the results of cognitive tests [18]. Improvement in cognitive abilities was observed after use of tramadol [13], inositol stabilized arginine [19] and polyphenols [17]. Snus, on the other hand, caused increased mental fatigue and mental load, and decreased perceived readiness [18]. Cocoa flavanols [15], alpha glycerophosphocholine, uridine-5ʹ-monophosphate and DHA [14] and choline/uridine DHA [16] did not affect cognitive abilities in the studies included in this review. Importantly, none of the tested substances improved all the cognitive abilities assessed, but only certain specific aspects. In all studies that showed a beneficial effect on cognitive functions, the participants were trained or highly trained athletes [13, 17, 19]. However, it is difficult to determine whether the level of training is relevant, as the other two studies included in the review involving trained athletes showed no effect on cognitive function [14, 15]. Impaired cognitive abilities, manifested as increased mental fatigue, were observed in recreationally active amateur football players [18]. Adverse reactions related to supplementation were reported in four studies [13, 17–19], whereas the remaining three [14–16] did not specify whether they gathered such information. Only four of the selected studies evaluated the impact of supplementation on physical performance [13, 14, 16, 18]. None of the studies observed an improvement in exercise capacity after the intervention. In none of the studies did the authors discuss the term ‘brain doping’. Sowinski et al. (2021) mentioned inositol stabilized arginine as a substance that ‘enhances neurotransmission’ [19], Decroix et al. (2016) referred to cocoa flavanols in the context of ‘cognitive enhancement’ [15], while Gibson et al. (2020) described enriched blackcurrant drink as a ‘nootropic brain drink,’ without specifying how they define nootropic effects [17].

**TABLE 2:** Characteristics of interventional studies included in the systematic review.

Author(s), year [ref]	Study design (washout time in days)	Dosage, substance and intervention duration	Number, sex and age of participants	Sport level	Cognitive aspects assessed & test performed	Cognitive Effect	Perfor-mance Effect	Adverse reactions (R/NR/NM)
Bejder et al. 2020 [13]	RCT (min. 4)	100 mg Tramadol	16M, 26 *± 5*	Highly trained 10 cyclists, 6 triathletes	Motor-cognitive abilities – 4 tasks	Average score for 2 tasks ↑ (P < 0.05) with Tramadol than placebo treatment, no effect for 2 remaining tasks	No effect	R

Bunn et al. 2018 [14]	RCT (2)	500 mg alpha glycerophosphocholine; 250 mg uridine-5’-monophosphate; 1500 mg DHA; acute ingestion 90 min. prior testing	20M, 21 ± 1	Trained training ≥ 3 d/wk for at least 12 weeks prior to the study	Verbal Memory, Visual Memory, Reaction Time, Processing Speed and Impulse Control, ImPACT	No effect	No effect	NM

Decroix et al. 2016 [15]	RCT (7)	900 mg of cocoa flavanol (in chocolate), 95 minutes prior to the test	12M, 30 *± 3*	Well trained (Performance level 3 accord. to De Pauw 2013)	Executive function – Stroop test	No effect	Not measured	NM

Deuster et al. 2002 [16]	RCT (7)	50 mg/kg b.m. choline	13M, 28 ± 2	Recreational $\dot{\text{V}}0_{2\max}$ (mL/kg/min) 54.4 ± 2.4	Choice reaction time, logical reasoning, visual vigilance, serial addition and subtraction, working memory, spatial memory, and decoding – MEL	No effect	No effect	NM

Gibson et al. 2020 [17]	RCT (10)	465 mg of total polyphenols and anthocyanins (155 mg), l-theanine (80 mg), pine bark extract (50 mg), and vitamin C (30 mg) per 100 mL · 100 mL of drink/ 7-day	19M; 4F, 28 *± 5*	Trained rugby league players (training 4.5 h/wk)	Mental clarity, mental stress, mental toughness, – Stroop Test, modified MTQ48	cognitive performance, Stroop test accuracy, perceived reliability, perceived nervousness control, and decreased perceived distraction.	Not measured	R

Morente-Sanchez et al. 2015 [18]	RCT (5)	1 g of snus (8 mg of nicotine)	18M, 23 *± 2*	Recreational Amateur football players	Perceived activation and perceived mental and physical fatigue & VAS	Negative effect: ↑ mental fatigue level and ↑ mental load, ↓ perceived readiness level	No effect	R

Sowinski et al. 2021 [19]	RCT (7–14)	1500 mg Inositol-enhanced bonded arginine silicate: inositol stabilized arginine (ASI) +inositol 100 mg, 15 minutes prior to the test	18M; 8F, 23 *± 5*	Not applicable experienced gamers (≥ 5 h gaming/week during past 6 months)	Reaction time, short term and working memory, sustained attention reaction time – PEBL battery, CBSRC, LTRT	↑ primary outcomes of reaction time and working memory	Not measured	R

CBSRCT – Cambridge Brain Sciences Reasoning and Concentration Tests, ImPACT – Immediate Post-Concussion Assessment and Cognitive Testing, LTRT – Light Tracking Reaction Test, MEL – Micro Experimental Laboratory software, MTQ48 – mental toughness assessment, NM – not mentioned, NR – not reported, PEBL – Psychology Experiment Building Language, R – reported, RCT – randomized controlled trial, VAS – visual analogue scale.

#### Case studies

Two case reports (Table 3) were included in the systematic review [20, 21]. All of them examined young men aged 25 [21] to 34 [20]. One study described the case of bodybuilder [20] and the other described the case of an amateur marathon runner [21]. The substances described were n-phenylacetyl-l-prolylglycine ethyl ester [21] and anabolic androgenic steroids [20]. Both studies described cases of excessive use of the above-mentioned substances. Neither study definitively indicated that supplementation was the main cause of hospitalization.

**TABLE 3:** Characteristics of case studies included in the systematic review.

Author(s), year [ref]	Study design	Substance	Dosage, duration of use	Sex, age of participant	Sport level	Symptoms at admission	Clinical outcome
Carvalho et al. 2016 [21]	Case report	Provigil (modafinil) and Noopept (n-phenylacetyl-lprolylglycine ethyl ester)	Not reported	M,25	Amateur marathon runner	Exertional heat stroke, tachypnoea and tachycardia, hypoglycaemia.	Acute liver failure

Choulerton et al. 2021 [20]	Case report	Anabolic androgenic steroids: testosterone, trenbolone	12 week period: 25 mg /d testosterone propionate, 50 mg/ trenbolone acetate followed by 4 weeks period: 20–40 mg/d tamoxifen and 25–50 mg/d clomiphene citrate	M,34	Recreational bodybuilder	A 1-day history of headache and right-sided visual loss	Ischaemic stroke

#### Market analysis

Thirty-four substances/extracts of plant/mushroom origin and 120 synthetic substances with potential pro-cognitive properties were identified. Among the synthetic substances, 26 were classified as ‘prohibited’ (Table 4), 49 as ‘non-prohibited’ (Table 5), and 45 were labelled as ‘unclear’, including 19 presumed to ‘meet WADA criteria’ (Table 6).

**TABLE 4:** Prohibited substances.

	Substance	Class on the WADA list
1	5-Methylhexan-2-amine (DMAA)	S6.B
2	1,3-Dimethylbutylamine (AMP-Citrate)	S6.B
3	Adrafinil	S6.A
4	Anastrozole	S4.1
5	Andarine (S-4)	S1.2
6	BPC-157	S0
7	Bromantan	S6.A
8	Centrophenoxine (Meclofenoxate)	S6.B
9	Clomifene (Clomid)	S4.2
10	Exemestane	S4.1
11	GW-0742*	S4.4
12	Hydrafinil	S6.A
13	Ibutamoren (MK-677)	S2.2
14	Ligandrol (LGD-4033)	S1.2
15	Meldonium	S4.4
16	Modafinil	S6.A
17	Octodrine (1,5-dimethylhexylamine; DMH)	S6.B
18	Ostarine (Enobosarm)	S1.2
19	Phenethylamine (Phenylethylamine; PEA)	S6.B
20	Phenylpiracetam (Fonturacetam; Carphedon)	S6.A
21	S-23	S1.2
22	Selegiline	S6.B
23	SR-9009	S4.4
24	Tamoxifen	S4.2
25	Testolone (RAD-140)	S1.2
26	Yk-11	S1.2

Note:

* GW-0742 is not explicitly listed on the WADA Prohibited List 2025, but it is included in the WADA 2022 Anti-Doping Testing Figures, published in April 2024

**TABLE 5:** Non prohibited substances.

No.	Substance	Source of information
1	5-Hydroxytryptophan (5-HTP)	NSF
2	Acetyl-L-carnitine (ALCAR)	NSF
3	Alpha-glycerophosphocholine (Alpha GPC; Choline alfoscerate)	NSF, IF
4	Beta ecdysterone	WADA monitoring program
5	Caffeine	WADA monitoring program
6	Cannabidiol (CBD)	WADA List
7	Chloroquine	GD
8	Choline bitartrate	NSF
9	Citicoline (CDP-Choline; cytidine diphosphate-choline; cytidine 5’-diphosphocholine)	NSF
10	Coenzyme Q10	NSF
11	Creatine	AIS
12	Dimethylaminoethanol (DMAE)	USADA
13	Galantamine	GD
14	Gamma aminobutyric acid (GABA)	NSF
15	Glutathione	GD
16	Hordenine	USADA
17	Huperzine A	USADA
18	L-alanyl-L-glutamine (Sustamine)	NSF
19	L-carnosine (β-alanyl-L-histidine)	AIS
20	Lithium orotate	NADA
21	L-theanine	NSF
22	L-threonate	NSF
23	L-tryptophan	NSF
24	L-tyrosine	NSF
25	Melatonin	NADA
26	Memantine	GD
27	N-acetyl-L-cysteine (NAC)	NADA
28	N-acetyl-L-tyrosine (NALT)	NSF
29	Omega 3-6-9	NSF
30	Phenibut	USADA
31	Phenylalanine	NSF
32	Phosphatidylserine	NSF
33	Picamilon	USADA
34	Piperine	AIS
35	Pipradrol (Morphodrol)	WADA monitoring program
36	Piracetam	GD
37	Sildenafil	NADA
38	Sulbutiamine	USADA
39	Synephrine	WADA monitoring program
40	Tadalafil	NADA
41	Taurine	NSF
42	Theobromine	NSF
43	Tianeptine	GD
44	Uridine	NADA
45	Vinpocetine	USADA
46	Vitamin B12 (Cobalamin)	NADA
47	Vitamin B6 (Pyridoxine)	NADA
48	Yohimbine	USADA
49	Zinc gluconate	NADA

AIS – Australian Institute of Sport; GD – Global DRO; IF – Informed Sport; NADA – National Anti Doping Agency of Germany; NSF – NSF Certified for Sport; USADA – United States Anti-Doping Agency.

**TABLE 6:** Substances with ‘unclear’ status, including presumed to ‘meet WADA criteria’.

	Substance	Similar chemical structure or similar biological effect(s) to (for presumed to ‘meet WADA criteria’):
1	1-(1- adamantylcarbonyl)proline (ACA)
2	3- amino-1-hydroxy-pyrrolidin-2-one (HA -966)
3	7,8-Dihydroxyflavone (7,8-DHF)
4	9-methyl-β- carboline (9-Me-BC)
5	Aceglutamide (Nauramina)
6	Adamantyl carbonyl proline
7	Agmatine
8	Aniracetam	4-phenylpiracetam (S6)
9	Berberine
10	Coluracetam (BCI-540; MKC-231)	4-phenylpiracetam (S6)
11	Cyclazodone	pemoline (S6)
12	Cycloserine
13	Dihydromyricetin
14	Emoxypine (Mexidol)
15	Epicatechin
16	Eutropoflavin (4’-dimethylamino-7,8-dihydroxyflavone; 4’-DMA-7,8-DHF)
17	Fasoracetam	4-phenylpiracetam (S6)
18	Fenozolone	pemoline (S6)
19	GTS-21 (DMXBA; DMBX-anabaseine)
20	IDRA-21
21	ISRIB
22	Methyl 3-(2-(benzyl(methyl)amino)ethyl)benzoate (PRL-8-53)	phenethylamine and its derivatives (S6)
23	Methyl-cypenamine
24	Methylliberine (Dynamine)
25	Nefiracetam	4-phenylpiracetam (S6)
26	N-ethyl-cypenamine	fencamfamin (S6)
27	N-methyl-cyclazodone	pemoline (S6)
28	Nooglutyl
29	Noopept	4-phenylpiracetam (S6)
30	NSI-189
31	Oleoylethanolamide
32	Oxiracetam	4-phenylpiracetam (S6)
33	P-Cl-phenylpiracetam (RGPU-95)	4-phenylpiracetam (S6)
34	Phenibut FAA
35	Phenyl-2-propylaminopentane (PPAP)	selegiline (S6)
36	Pramiracetam	4-phenylpiracetam (S6)
37	Pregnenolone	testosterone precursor (S1)
38	Pterostilbene
39	Sarcosine
40	Selank
41	Semax	tetracosactide (S2)
42	Sunifiram	4-phenylpiracetam (S6)
43	Theacrine
44	Thozalinone	pemoline (S6)
45	Zylofuramine	etilamfetamine (S6)

### DISCUSSION

In most sports disciplines, there is a need for improvement in physical and mental performance. These factors, apart from strength and endurance, include sensory perception and processing and multiple aspects of executive function, such as dual tasking and response inhibition [22]. Recent advances in neuroscience and neuropharmacology also support enhancing muscle strength and pushing the limits of endurance by using technologies that change brain activity [23]. Therefore, brain doping or neuro-doping might be defined as the use of substances (pharmaceuticals) or technologies (electrical brain stimulation) to improve concentration and memory, including motor learning, or reduce fatigue [3, 5, 7–9, 22].

There is interest in the area of brain doping among athletes. Scientific interest in research on brain doping has also grown significantly, best illustrated by the fact that a PubMed search for ‘brain doping’ or ‘doping for the brain’ shows 48 results until 2003 and 594 hits in 2003–2023. Despite hundreds of studies, it is challenging to specify well-defined and described substances that could contribute to improving cognitive abilities in sports. Only 9 research studies have investigated the current knowledge on substances that have or may affect the brain at the cognitive level in the context of sports. For this reason, we reviewed results from the PubMed database and substances found on websites and Polish e-shops with dietary supplements.

The systematic review of the literature we conducted to find evidence of the beneficial effects of substances advertised as brain doping on physical performance or the improvement of athletic performance did not yield satisfactory findings that could significantly resolve our doubts regarding the status of the considered compounds. In the studies we ultimately included, the following substances or extracts were described: snus (nicotine), tramadol, inositol-enhanced bonded arginine silicate, cocoa flavanol, Ārepa supplement (blackcurrant-based nootropic-drink containing pine-bark and l-theanine), alpha glycerophosphocholine (alpha-GPC), uridine-5ʹ-monophosphate, DHA, choline, taurine, testosterone, trenbolone, modafinil and Noopept (N-phenylacetyl-l-prolylglycine ethyl ester). Only L-theanine, alpha-GPC, and taurine appeared as compounds of supplements in the offerings of online stores associated with ‘brain doping’. These substances have been classified as non-prohibited. L-theanine, a non-proteinogenic amino acid naturally found in tea, is claimed to alleviate anxiety and stress, as well as supporting cognitive function, and there is some evidence suggesting that it could work synergistically with caffeine [24, 25]. It can pass through the blood-brain barrier and is believed to offer neuroprotective benefits. The results of studies involving athletes, however, are scarce and inconclusive regarding the impact of L-theanine on physical and/or cognitive performance [25]. Alpha-GPC is a choline-containing phospholipid involved in neurotransmitter synthesis. It is commonly used as a supplement to improve cognitive function, prevent cognitive decline [26], and boost power output in sports performance. The procognitive effects of alpha-GPC have been confirmed in rodents and in patients with neurological conditions, but its effectiveness in the athletic population still needs to be validated through research [27]. Taurine is a non-proteinogenic amino acid, especially abundant in skeletal muscle. Evidence on whether taurine improves sports performance is limited and inconclusive, thus requiring further research [28, 29]. Our systematic review also demonstrated that the beneficial effect of the studied substances on cognitive aspects was present only in athletes at the trained or highly trained level. This result differs from those reported by other authors; however, due to the limited data available, we were not able to confirm this with full certainty. In general, amateur athletes tend to derive greater benefits from supplementation than individuals with a high level of training, as they start from a lower baseline [30]. Elite athletes, due to years of training, have already developed specific physiological adaptations. For example, amateur athletes may experience greater cognitive benefits from creatine supplementation than highly trained individuals, likely because amateurs have lower baseline levels of this substance in their bodies [31].

The study aimed to identify compounds promoted and sold as ‘brain doping’ and assess their status concerning anti-doping regulations and scientific literature. Among the 120 substances identified in dietary supplements, 22% were classified as prohibited, 41% as non-prohibited, 37% had an unclear status, but for 16%, it was hypothesized that they meet WADA doping criteria.

Determining the status of some of the substances with potential pro-cognitive effects was straightforward, as they are doping agents (Table 4), listed on the WADA list, or are included in the Monitoring Program, and according to the WADA rules, they are not considered prohibited substances (Table 5). As an illustration, for many years, following the opinion of experts, according to which ecdysterone met the criteria for recognition as doping [32, 33], the Polish Anti-Doping Agency recommended athletes to refrain from using supplements with this agent. When in 2020 WADA included ecdysterone in their Monitoring Program, it became clear that this substance is not currently considered doping. It is a basic right of an athlete to be clear about the status of a substance that is an ingredient in any pharmaceutical drug or dietary supplement. Unfortunately, in many cases, it is impossible to determine the status of a particular substance, i.e. whether it is prohibited or permitted. The WADA Prohibited List still has an open character. Despite the numerous examples of prohibited substances or methods in particular classes, some additional agents, which are not included in the list but have ‘a similar chemical structure or similar biological effect(s)’, may be considered as doping. Such an approach allows for the initiation of investigative procedures when an athlete uses new pharmacological substances, including substances specifically intended for doping purposes [34]. This also leads to numerous challenges in identifying which substances are currently permitted and which are prohibited in sports. Additionally, WADA’s approach to this matter lacks consistency. For instance, in 1999, during the Manfred Donike Workshop on Dope Analysis, it was suggested that midodrine, a vasopressor and antihypotensive medication, should be added to the doping list [35]. Although in databases such as Global DRO and NADAmed this substance had a prohibited-in-competition status, WADA only confirmed this in 2024, placing midodrine among the examples of class S6 stimulants on the 2025 List of Prohibited Substances and Methods [1]. In recent years, some substances (e.g. meldonium, tramadol) have been regarded as doping agents only after inclusion on the prohibited list following an earlier announcement. In parallel, some athletes were disqualified for using substances (e.g. methylhexanamine, higenamine, ractopamine) before they were listed among the examples of doping agents.

In the current study, some substances were classified as ‘not prohibited’ even if they were not explicitly described as permitted by sources such as Global DRO or NADAmed, but had been evaluated at least indirectly. Examples include compounds such as dimethylaminoethanol (DMAE), huperzine A, vinpocetine, and yohimbine. In the Supplement Guide available on the USADA website, the composition of an energy drink is presented, listing these substances. However, they were not marked as prohibited, unlike other ingredients in the supplement, such as 2-aminoisoheptane, higenamine, and phenethylamine. We also included hordenine, phenibut, and vinpocetine in the ‘not prohibited’ group, as they were listed on the USADA website under the category ‘Ingredients that WADA does not prohibit (you will not incur an anti-doping violation for using these ingredients).’ Interestingly, DMAE, under the name deanol, was listed as an example of a doping agent in the stimulant class by the Anti-Doping Authority of the Netherlands in 2007, and hordenine is considered a doping agent in horses and is also prohibited by the National Collegiate Athletic Association (NCAA). Piperine was also included in the ‘not prohibited’ group, based on a description by AIS found in the characterization of curcumin from group B, which states, ‘curcumin is an unstable compound and its bioavailability is very poor in its unmodified form. Companies have formulated supplements with piperine (pepper extract), turmeric oil, or soy lecithin, and particle size to improve absorption.’

It should be emphasized that the scientific literature also raises doubts about the substances we classified as ‘not prohibited’ in our work. For example, Kennedy (2021), in a systematic review of theobromine and theophylline, stated that although the studies showed contradicting results and/or insufficient data to draw solid conclusions, it appears both drugs have the potential to enhance performance and could be considered for inclusion on the WADA banned list [36]. However, it would probably be difficult to implement because theobromine is not only a fairly popular ingredient in dietary supplements, but it is also the predominant compound present in chocolate.

The issue of the open nature of the WADA Prohibited List is particularly relevant to dietary supplements, the use of which is common in sports. Between 40% and 100% of athletes typically use supplements, depending on the type of sport, level of competition, and the definition of supplements [37]. Doping control forms reflect this trend, with every second form reporting the use of at least one supplement [38]. Notably, 28% to 38% of these supplements carry the risk of leading to unintentional doping [39]. Ultimately, it is estimated that between 6.4% and 8.8% of doping cases are associated with supplement use [40].

Undoubtedly, the substances which were classified under the ‘unclear status’ category may raise the most doubts in light of anti-doping regulations. In some cases, we added the note ‘meets WADA criteria’ due to their chemical structure being similar to substances on the WADA list or their biological effects being similar (as they are presented in online offers as having effects comparable to specific doping agents). This group includes, among others, derivatives of piracetam, which is an authorized pharmaceutical in some countries in Europe but has not been approved as a drug by the US FDA [41]. Piracetam has ‘non-prohibited’ status (NADA, GlobalDro), but the Banned Substances Control Group (BSCG), a third-party certification and testing provider of dietary supplements, includes nine racetams on its Dietary Supplement Ingredient Advisory List [41]. Because piracetam derivatives are often presented as more potent than piracetam and are not licensed for medical use, however, we have classified these substances as similar to 4-phenylpiracetam. This substance, known as carphedon, was unofficially produced in Russia by the Research Institute of Cosmic Medicine and used as a stimulant and ‘stress protector,’ and at a 100 mg dose was shown to support exercise performance. Carphedon became the first nootropic prohibited in sport in the 1998 by IOC [41].

The group also includes alkaloids structurally similar to caffeine, e.g. theacrine and methylliberine. Not enough research exists to confirm that theacrine may be useful as a supplement. There are more abundant data on methylliberine, which is an isolate of coffee beans, tea, cola nuts, guarana, cocoa, and yerba mate [42]. According to La Monica et al. (2023), methylliberine did not enhance cognitive function but did significantly enhance healthy subjects’ perceptions of energy, concentration, motivation, and mood over time [43]. Notably, this substance is included in the Therapeutic Goods Act, part of the Australian Government Department of Health, which is a legislative instrument to help protect Australian consumers from the unsafe use of certain sports supplements [44]. The instrument declares certain sports supplements (those that include higher-risk ingredients or are in the form of a tablet, pill or capsule) to be therapeutic goods, ensuring that they are appropriately regulated as medicines. Methylliberine is classified as a relevant substance, which means this substance is not included in a schedule to the current Poisons Standard or expressly identified on the WADA Prohibited List. According to the Advisory Committee for Novel Foods (ACNF), there is currently a lack of robust safety assessment data on methylliberine, its effects on the human body, and its synergistic effect on the action of other stimulants, such as caffeine.

In the era of modern sports, both professional and amateur athletes are under huge pressure to improve performance rapidly and to be consistent in delivering results. This pressure drives many to boost their performance using additional methods. It is worth noting that products sold as supplements that improve cognition are often used by young people (especially in the context of the massive popularity of e-sports). It should be underlined that substances defined as ‘neuro-doping’ or ‘brain doping’ are well advertised, but this marketing is not supported by scientific evidence. Defining and classifying substances with a potential beneficial effect on improving cognition will be essential. One can absolutely agree with the conclusion of the article by Jędrejko et al. (2023) that nootropic dietary supplements in general represent a growing category of pharmaceutically active substances that is likely to challenge regulators and anti-doping authorities in the future as ingredient options continue to expand [41]. In line with this trend, tesofensine, also sold online as a dietary supplement, has recently been classified as a doping agent on the WADA 2025 prohibited list. It is a triple monoamine re-uptake inhibitor with an affinity for dopamine (DAT), serotonin (SERT), and norepinephrine (NET) transporters. This nootropic agent also stimulates cholinergic neurons of the prefrontal cortex and hippocampus [45]. More substances with nootropic effects can be expected to appear on the WADA Prohibited List in the future.

Here, we systematically reviewed existing scientific literature using keywords selected by authors that might be considered a limitation of the study. It should also be mentioned that the current review focuses on one side of the evidence, which is identifying substances with proven beneficial effects on sports performance, and due to the small number of articles discussed, may not completely answer all clinically or practically relevant questions. Finally, among the substances identified based on the market analysis, most of them showed an ambiguous status, which may render these findings difficult to incorporate into practice. Many products offered in online stores as dietary supplements are illegal in terms of national or international regulations. They may contain substances that are simultaneously applied in medicinal products as defined in the pharmaceutical law, which as a matter of principle is not applicable to dietary supplements. Sometimes the same products are offered in one place as dietary supplements, and in others as chemical reagents or standards for research only. Given the open-ended nature of the WADA Prohibited List, the legal maxim ‘everything that is not forbidden is allowed’ is insufficient to prevent a positive doping test result. It is important to keep in mind the principle of limited trust, i.e., ‘what is not forbidden does not necessarily mean it is allowed’. If someone has doubts about dietary supplements marketed as brain doping, they should primarily follow the recommendations of scientific societies and/or institutions in the field of professional sports.

### CONCLUSIONS

In conclusion, the term ‘brain doping’ currently serves more as a marketing tool than as a concept describing a group of substances with scientifically proven cognitive effects. The group of substances marketed to consumers as ‘brain doping’ includes substances prohibited in sports, as mentioned on the WADA Prohibited List, and those permitted for use by athletes or of unclear status. Although the list of prohibited substances and methods is updated at least annually, its open-ended nature continues to pose challenges in definitively determining the status of many substances found in dietary supplements or medications, which may lead to unintentional violations of anti-doping rules.

## Supplementary Material

„Brain doping” substances: prohibited or not in sports?
