# Supplementary material for: „Brain doping” substances: prohibited or not in sports?
Source: Biol Sport. 2025 May 14;42(4):189–201. doi: 10.5114/biolsport.2025.150047 (PMC12492343; doi:10.5114/biolsport.2025.150047)
Supplement: „Brain doping” substances: prohibited or not in sports? [file JBS-42-4-56026-s1.pdf]

TABLE S1. The list of ingredients/supplements from the botanicals category.

Acanthopanax senticosus	Green oat (Avena sativa)
Ashwagandha	Hypericum perforatum (St John's wort)
Asparagus Racemosus (Shatavari)	Inonotus obliquus (Chaga)
Bacopa monnieri	Lion's mane
Cocoa extract	Mucuna pruriens (Velvet bean)
Cannabis	Passiflora incarnata (Passionflower)
Convolvulus Pluricaulis	Paullinia cupana (Guarana)
Cordyceps militaris	Pausinystalia yohimbe
Crocus sativus (Saffron)	Piper methysticum (Kava)
Curcuma longa (Curcumin)	Polygala tenuifolia (Yuan zhi)
Evolvulus glomeratus (Dwarf morning glory)	Ptychopetalum olacoides (Muira puama)
Ganoderma lucidum (Reishi)	Rhodiola rosea
Ginger	Sceletium tortuosum (Kanna)
Ginkgo biloba L	Schisandra shinensis
Ginseng (Panax ginseng C.A. Meyer)	Shilajit mumio
Gotu kola (Centella asiatica L)	Tongkat ali
Green matcha	Trifolium pratense L (Red clover)
